# Medical Fees and the Public

**Published:** 1909-10-23

**Authors:** 


### Medical Fees and the Public.

An enterprising American contemporary has recently devoted considerable attention to the question of medical fees. This activity has apparently arisen from a consideration of the fact that there is a movement on foot in Paris and Vienna, among the *internes* attached to the larger hospitals, to agitate for an increase of salary. It is an undoubted fact that the junior staff of hospitals, all the world over, is paid at a rate out of all proportion to what it accomplishes. At our London hospitals for example, many recently qualified men slave devotedly on behalf of their hospitals and patients at a pittance which would evoke the commiserating contempt of a metropolitan office boy. In the provinces, where competition is less keen, and compensatory non-financial advantages are less conspicuous, members of the resident staff are proportionately better paid, but even in the provinces there is room for improvement. On the Continent, medical men offer their services to the public, at least so far as hospitals are concerned, at a valuation which is certainly very much lower than that which an English doctor, not to speak of an American, would place upon his professional skill. In general, too, the scale of Continental fees is lower than the English or American scales, and in some countries, as in Germany and in Italy, it is to some extent regulated by law. In England, although the public takes it for granted that the profession as a whole adheres to scales generously drawn up for them by the compilers of certain popular annuals, there is no fixed or established scale of charges. Nor do we think it advisable that there should be. It is impossible to place a definite monetary value upon professional services, either in medicine or in law, and it would be foolish to attempt to do so. In both professions, practitioners must use their own discretion, and must attempt to obtain what they can get. Success, and success only, can regulate the fee—not, be it understood, success in individual cases, but professional success, which means eminence and a substantial increase in the money-earning capacity of the practitioner. To endeavour to bring the public under the sway of a huge professional trades union—and that, it seems to us, will be the inevitable outcome of an attempt to fix a maximum rate of fees—will be prejudicial to the best interests of the profession as well as of the public. Taken as a whole, fees in England are not lower than they should be. In many cases they are far higher than, to take a comparative view, it is necessary for them to be. We do not of course refer to such things as contract practice, club practice, or appointments on resident staffs, in all of which appears an element of that most horrible of collectivist dangers, trade-sweating, but solely to the average general practitioner's scale of fees. It is unnecessary to go into the question more fully in a professional paper, and we believe we have the authority of so eminent an expert in the sister profession, the late Lord Chief Justice Russell of Killowen, for holding that it is both unnecessary and impertinent to discuss it in the lay press.

